# In Silico and in vitro Characterization of *Lactobacillus acidophilus* Bacteriocins as ROR-1-Targeted Therapeutics in Breast Cancer

**DOI:** 10.5812/ijpr-163509

**Published:** 2025-12-22

**Authors:** Sara Soheili, Shekufe Rezghi Barez, Seyed Davar Siadat, Seyed Hossein Hejazi, Hamid Abtahi

**Affiliations:** 1 Molecular and Medicine Research Center, Arak University of Medical Sciences, Arak, Iran; 2 Department of Clinical Biochemistry, School of Pharmacy and Pharmaceutical Sciences, Isfahan University of Medical Sciences, Isfahan, Iran; 3 Department of Mycobacteriology and Pulmonary Research, Pasteur Institute of Iran, Tehran, Iran; 4 Department of Parasitology, Isfahan University of Medical Sciences, Isfahan, Iran

**Keywords:** Molecular Dynamics, *Lactobacillus acidophilus*, Bacteriocins, Intestinal Microbiota, Molecular Docking, Simulations

## Abstract

**Background:**

The human microbiota plays a crucial role in maintaining host health and is involved in various illnesses, including cancer. The intestinal microbiota has been identified as a factor in the development of colorectal carcinoma and breast cancer (BC).

**Objectives:**

This study investigated the anticancer properties of bacteriocins produced by *Lactobacillus acidophilus*, specifically their interaction with receptor tyrosine kinase-like orphan receptor 1 (ROR-1), a protein involved in aggressive BC subtypes.

**Methods:**

Employing sophisticated computational methodologies, encompassing molecular docking and molecular dynamics (MD) simulations, this research elucidated the dynamic interactions and binding strengths of four distinct bacteriocins — Acidocin A, Acidocin B, Acidocin 8912, and Acidocin J1132β — with the ROR-1 receptor. This observation was substantiated by consistent hydrogen bond formation and low root mean square deviation (RMSD) values throughout the simulation period. Furthermore, the biological activity of crude acidocins was evaluated on the ROR-1-Src signaling axis in Michigan Cancer Foundation-7 (MCF-7) and MDA-MB-231 cell lines utilizing Western blot analysis.

**Results:**

The findings of this research demonstrate that Acidocin A exhibits promise as a prospective therapeutic intervention directed at ROR-1 in BC. Furthermore, the synergistic application of molecular docking, MD simulations, and molecular mechanics/Poisson-Boltzmann surface area (MM/PBSA) free energy calculations yielded a thorough elucidation of the underlying interaction mechanisms. The assessment of protein expression levels indicated a significant downregulation of the ROR-1-Src signaling pathway following treatment with Acidocins.

**Conclusions:**

This research highlights the potential of bacteriocins in cancer treatment and adds to evidence linking the microbiota to cancer, establishing new pathways for anticancer therapies from microbial sources.

## 1. Background

Breast cancer (BC) represents the most commonly diagnosed malignancy and a leading cause of cancer-related mortality globally (1). Research has suggested a link between an imbalance in gut bacteria (microbiota) and BC, similar to its connection with colorectal cancer (2). The microbiota exerts influence over cellular and organ physiology through the production of metabolites and proteins, notably bacteriocins, which possess the capacity to translocate into the bloodstream and thereby access distal organ sites (3, 4). The majority of bacteriocins are characterized as low molecular weight peptides, typically below 10 kDa (5). Bacteriocins are of research interest due to their potential antineoplastic properties (6). They can modulate cellular processes, including inflammatory and immune responses (3). Receptor Tyrosine Kinase-Like Orphan Receptor 1 (ROR-1) plays a critical role as an immunological modulator within BC cells that are associated with the microbiota (7). Notably, ROR-1 exhibits elevated expression levels in triple-negative BC and other aggressive BC subtypes, a phenomenon that correlates with poorer clinical prognoses (2, 8). Consequently, ROR-1 has been identified as a potential target for tumor-directed therapeutic interventions (9). The Wnt-related integration site (Wnt) signaling axis plays a significant role in the advancement of cancer, notably in processes such as tumor metastasis, cellular proliferation, and invasive behavior (10). The convergence of these signaling pathways can promote oncogenic mechanisms. Given its integration within the ROR1-Src-Wnt signaling pathway, ROR-1 is implicated in influencing the pathogenesis of BC (11). Src, a non-ROR1, functions as a substrate for ROR-1. Upon ROR-1 activation, it can phosphorylate and consequently activate Src, thereby enabling the further transduction of downstream signaling cascades (12).

To comprehensively understand the interplay between bacteriocins and cancer cell receptors, such as ROR-1, sophisticated computational methodologies, including molecular dynamics (MD) simulations and molecular docking, were deemed essential. Molecular docking serves as a robust computational technique for predicting the binding affinity and spatial arrangement of bacteriocins at their designated receptor targets. This method furnishes an atomic-level depiction of potential intermolecular interactions. Meanwhile, MD simulations provide a dynamic approach, enabling observation of the time-dependent evolution of these interactions and elucidating the stability and conformational transitions that occur over the simulation period. The synergistic application of these methodologies not only enhances our mechanistic understanding at the molecular level but also presents substantial potential for the development of innovative therapeutic interventions in the context of cancer treatment (13). Recent studies suggest that *Lactobacillus acidophilus* may enhance inflammatory and immune responses in BC by manipulating gut microbiota (14).

## 2. Objectives

This research investigated the interactions between acidocins produced by *L. acidophilus* and ROR-1, a receptor involved in BC. Computational methods were used to study the interaction between specific acidocins and ROR-1. The study's findings may lead to new therapeutic interventions, but further research is needed to confirm their effectiveness and understand the underlying mechanisms.

## 3. Methods

### 3.1. Peptide Preparation

The potential anticancer properties of four Acidocins derived from *L. acidophilus* were investigated (15). Given the absence of crystallographic structures for acidocin 8912, acidocin J1132 β, and acidocin A (Table 1), their sequences were retrieved from the GenBank database. These sequences were then utilized for computational three-dimensional (3D) structure prediction employing the Swiss-Model server (https://swissmodel.expasy.org) (16). In contrast, Acidocin B was the only bacteriocin in this study with a previously resolved structure, accessible through the Research Collaboratory for Structural Bioinformatics (RCSB) Protein Data Bank (PDB code: 2MWR). To evaluate the reliability of the computationally generated models, Ramachandran plot analysis was conducted. Subsequently, to identify the most energetically favorable conformation for all the Acidocins, 50 nanosecond (ns) MD simulations were performed using the GROMACS 2022.6 software package (17).

**Table 1.** Bacteriocin Reference ID and Sequences

Bacteriocin	Reference (GenBank/PDB ID)	Sequence/3D Structure
**Acidocin A**	BAA07120.1	MISMISSHQKTLTDKELALISGGKTYYGTNGVHCTKK SLWGKVRLKNVIPGTLCRKQSLPIKQDLKILLG WATGAFGKTFH
**AcidocinB**	2MWR	3D Structure
**Acidocin8912**	BAA07737.1	MISSHQKTLTDKELALISGGKTHYNAWKSLWKGFWESLRYTDGF
**Acidocin J1132 β**	AAB49524.1	GNPKVAHCASQIGRSTAWGAVSGA

### 3.2. Molecular Docking Studies

The most energetically stable conformation of the bacteriocins, as determined through MD simulations, was employed as the ligand in the hybrid docking (HDOCK) server (http://hdock.phys.hust.edu.cn/), an integrated platform designed for robust and rapid protein-protein docking (18). For receptor selection, the Kringle domain of human ROR-1 was chosen due to its availability as a crystal structure with the PDB ID: 7TNG and a high resolution of 1.40 Å, which was the most favorable among the existing crystallographic data for ROR-1. The 3D structure of this domain was prepared and subsequently submitted to the HDOCK server, with all other parameters set to their default values. Following the docking procedure, the resulting bacteriocin-ROR-1 complexes exhibiting the lowest docking energy were subjected to further MD simulations for more detailed analysis.

### 3.3. Molecular Dynamics Simulation Studies

The behavior of ROR-1 in the presence of Acidocins was investigated using MD simulations with the GROMACS 2022.6 software package (17). The initial configuration for the simulations was derived from the lowest energy pose obtained from molecular docking studies. The acidocin peptides and the ROR-1 protein were parameterized using the AMBER99SB force field (19). The simulation system was solvated in a cubic box filled with transferable intermolecular potential 3-point (TIP3P) water molecules, and the overall charge of the system was neutralized by the addition of counterions. Periodic boundary conditions were applied in all spatial dimensions. The system's initial energy state underwent minimization utilizing the steepest descent algorithm. Subsequently, canonical (NVT) ensemble equilibration was performed employing the Berendsen thermostat (with a coupling constant of 0.1, 100 ps at a temperature of 300 K) (20). This was followed by an isobaric-isothermal (NPT) ensemble equilibration maintained with the Parrinello-Rahman barostat (a coupling constant of 5.0 ps, 1 ns at a pressure of 1 bar) (21). The simple exact transformation to linearize equations (SETTLE) algorithm (22) was implemented to maintain hydrogen bond constraints, while the linear constraint solver (LINCS) algorithm (23) was used to enforce the geometry of water molecules. Furthermore, electrostatic interactions were calculated using the particle mesh Ewald (PME) method (24). The simulation was executed over 100 ns, with trajectory frames saved at intervals of two femtoseconds. Subsequent analysis and visualization of the generated trajectories were performed using certain software including xmgrace, Discovery Studio 2016, and visual MD (VMD) (25). These analyses revealed notable conformational alterations and elucidated the influence of acidocin on ROR-1.

### 3.4. Molecular Mechanics-Poisson Boltzmann Surface Area Binding Free Energy

The binding free energy of protein-peptide complexes was quantitatively assessed in this research using the molecular mechanics/Poisson-Boltzmann surface area (MM/PBSA) method, as implemented in the gmx_MMPBSA program (26). To determine the total binding free energy (ΔGTOTAL), the final 1 ns of the MD simulation trajectories for each complex was analyzed. The implicit solvent model was parameterized with a solvation parameter (igb) of 5, and a physiological salt concentration of 0.154 M was incorporated. The dielectric constants for the solute interior and the solvent were set to their default values of 1.0 and 80.0, respectively.

The MM/PBSA calculation process is governed by the following equations:

Equation (1):


$$\Delta G=G_{complex\;}-\;[G_{receptor\;}+\;G_{ligand}]$$

This equation computes the binding free energy of the ligand, receptor, and complex, based on trajectory frames extracted from the final 1 ns of the MD simulation.

Equation (2):


$$\Delta SASAi\;={SASA}_{i}^{freeform}-{SASA}_{i}^{bound}\;$$

This equation determines the difference in solvent-accessible surface area (SASA) between the unbound and bound states of the components.

The energy components used in the calculation of ΔG are detailed as follows:

Equation (3):


$$\;{\Delta G}_{binding}=\Delta H\;-\;T\Delta S$$

Equation (4):


$$\Delta H\;={\Delta G}_{GAS\;}+\;{\Delta G}_{SOLV}$$

Equation (5):


$${\Delta G}_{GAS\;}={\Delta E}_{bonded\;}+\;{\Delta E}_{nonbonded\;}=\;({\Delta E}_{bond\;}+{\Delta E}_{angle\;}\;+{\Delta E}_{dihedral}\;)\;+\;({\Delta E}_{ele\;}+{\Delta E}_{vdW}\;)$$

Equation (6):


$${\Delta G}_{SOLV\;}={\Delta E}_{polar\;}+{\;\Delta E}_{non-polar\;}=\;{\Delta E}_{PB/GB}+{\Delta E}_{non-polar}$$

These formulations follow the standard description provided in the AMBER manual (https://ambermd.org).

This passage elucidates the thermodynamic components that contribute to the enthalpy change (ΔH) in molecular binding events. The enthalpy change is presented as a composite of the solvation free energy (ΔGSOLV) and the gas-phase energy (ΔGGAS). Furthermore, the term TΔS quantifies the entropic contribution to the overall binding free energy. ΔGGAS is further dissected into bonded interactions (ΔEbond, ΔEangle, and ΔEdihedral) and nonbonded interactions (ΔEele and ΔEvdW) forces. In the same way, ΔGSOLV is made up of the nonpolar solvation energy (ΔEnon-polar) and the polar solvation energy (ΔEpolar). The polar part of the binding free energy is determined through the Poisson-Boltzmann (PB) approach.

Preliminary investigations were conducted to ascertain the requisite simulation duration for complete dissociation of a solitary peptide from the protein structure. These initial experiments indicated that peptide detachment typically occurred within a temporal range of 300 to 400 ps. Consequently, the steered MD (SMD) simulation time was established at 500 ps to guarantee complete separation of the peptide from the protein molecule. Subsequent to the completion of the SMD simulations, a comparative analysis was performed to evaluate the binding affinities of the peptides. The peptide exhibiting the highest binding affinity was identified as a potential inhibitor of the target protein.

### 3.5. Biological Studies

#### 3.5.1. Microbial and Cell Culture

Pure probiotic strains, specifically *L. acidophilus* and *Bifidobacterium bifidum*, were procured from the microbial repository of the Iranian Scientific and Industrial Research Organization (designated as PTCC:1643 and PTCC:1644, respectively). To facilitate the acquisition of crude bacteriocin (CA), lyophilized cultures of *L. acidophilus* and *B. bifidum* were initially cultivated on de Man, Rogosa, and Sharpe (MRS) agar medium and incubated at 37°C for a duration of 24 hours. Subsequently, a single colony of the target bacterium was transferred under aseptic conditions using a sterile swab and inoculated into liquid MRS culture medium supplemented with 0.05% sterilized L-cysteine. Given the anaerobic nature of these two bacterial species, the microbial cultivation was performed under anaerobic conditions. Following a 72-hour incubation period, the cellular components were separated from the supernatant by centrifugation at 12,000 g for 10 minutes. Subsequently, the pH of the resulting supernatant was adjusted to 7.0 ± 0.1 using 0.1 M sodium hydroxide (NaOH) and hydrochloric acid (HCl). This solution was then subjected to sterile filtration using syringe filters with a pore size of 0.22 µm and subsequently purified through precipitation with 70% ammonium sulfate at 4°C under agitation for 30 minutes at 5°C. To precipitate the protein, the resultant supernatant underwent a subsequent centrifugation step at 6000 × g for 15 minutes. The resulting pellet was then re-suspended in 50 mL of phosphate-buffered saline (PBS) to yield a stock solution of CA. This preparation was stored at -20°C in test tubes for subsequent analyses (27, 28).

The Michigan Cancer Foundation-7 (MCF-7) (NCBI No.: C135) and MD Anderson-Metastatic Breast-231 (MDA-MB-231) (NCBI No.: C578) cell lines were procured from the National Gene Bank and Biological Resources of Iran. These cell lines were subsequently cultured in Dulbecco's Modified Eagle's Medium (DMEM; Gibco, USA), supplemented with 1% penicillin-streptomycin (PenStrep; Gibco, USA) and 10% fetal bovine serum (FBS; Gibco, USA). Cell incubation was maintained at 37°C with a 95% humidified atmosphere containing 5% carbon dioxide (CO_2_) (29). We utilized untreated MCF-7 and MDA-MB-231 cell lines as controls.

#### 3.5.2. Cytotoxicity Assay (Inhibitory Concentration Determination)

The cytotoxic effects of extracts derived from *L. acidophilus* and *B. bifidum* were evaluated on MCF7 and MDA-MB-231 cell lines using the [3-(4,5-dimethylthiazol-2-yl)-2,5-diphenyltetrazolium bromide] (MTT) assay. Initially, cells were seeded in 96-well plates at a density of 1 × 10^5^ cells per well and cultured in DMEM medium supplemented with 10% FBS. Following a 24-hour incubation period, the cells were subjected to treatment with crude acidocin at varying concentrations, employing a twofold serial dilution method. The protein concentrations of these dilutions, prepared in 1% DMEM, ranged from 5 μg/mL to 10 mg/mL and were applied for 48 hours in triplicate (27). The assay was conducted following a previously established protocol. Subsequent to the 48-hour treatment, 20 µL of MTT solution was introduced into each well and incubated for an additional 4 hours. Finally, the supernatant was aspirated, and 100 µL of dimethyl sulfoxide (DMSO) was added to each well. Absorbance was then measured at a wavelength of 570 nm using an ELISA reader (30).

#### 3.5.3. Western Blot Assay

The procedures for whole-cell lysate preparation and Western blot analysis were conducted as previously detailed (31). Following a 48-hour treatment period, cells were harvested and lysed using radio-immunoprecipitation assay (RIPA) buffer at a ratio of five volumes of buffer per unit volume of packed cells. Subsequently, the resulting lysate underwent centrifugation at 14,000 × g for 10 minutes at 4°C. Protein concentration in the supernatant was quantified utilizing the Bio-Rad protein assay kit (Bio-Rad Laboratories, Inc.). Subsequently, a total protein mass of 60 µg from the cell lysate was subjected to electrophoretic separation on a sodium dodecyl sulfate-polyacrylamide gel electrophoresis (SDS-PAGE) gel and subsequently transferred onto a polyvinylidene difluoride (PVDF) blotting membrane (Amersham Pharmacia Biotech, Buckinghamshire, UK) employing a Mini Trans-Blot^®^ Cell Module. The membrane was blocked via overnight incubation at 4°C using a 5% (w/v) solution of non-fat dried milk in Tris-buffered saline containing 0.1% Tween-20 (TBST). Subsequently, the membrane was incubated overnight at 4°C with the following primary antibodies: A mouse monoclonal anti-c-Src (B-12) (1:200 sc-8056), a rabbit recombinant monoclonal c-Src phospho Y418 antibody (1:50; ab40660), and a mouse monoclonal anti-β-actin (1:200; sc-517582). Following six washes with TBST, the membrane was incubated at ambient temperature for 60 minutes. Subsequently, protein detection was performed using the Thermo Scientific Pierce ECL Western Blotting Substrate.

## 4. Results

### 4.1. Preparation and Optimization of Acidocin Peptides Produced Stable Structural Model

**Figure 1.**
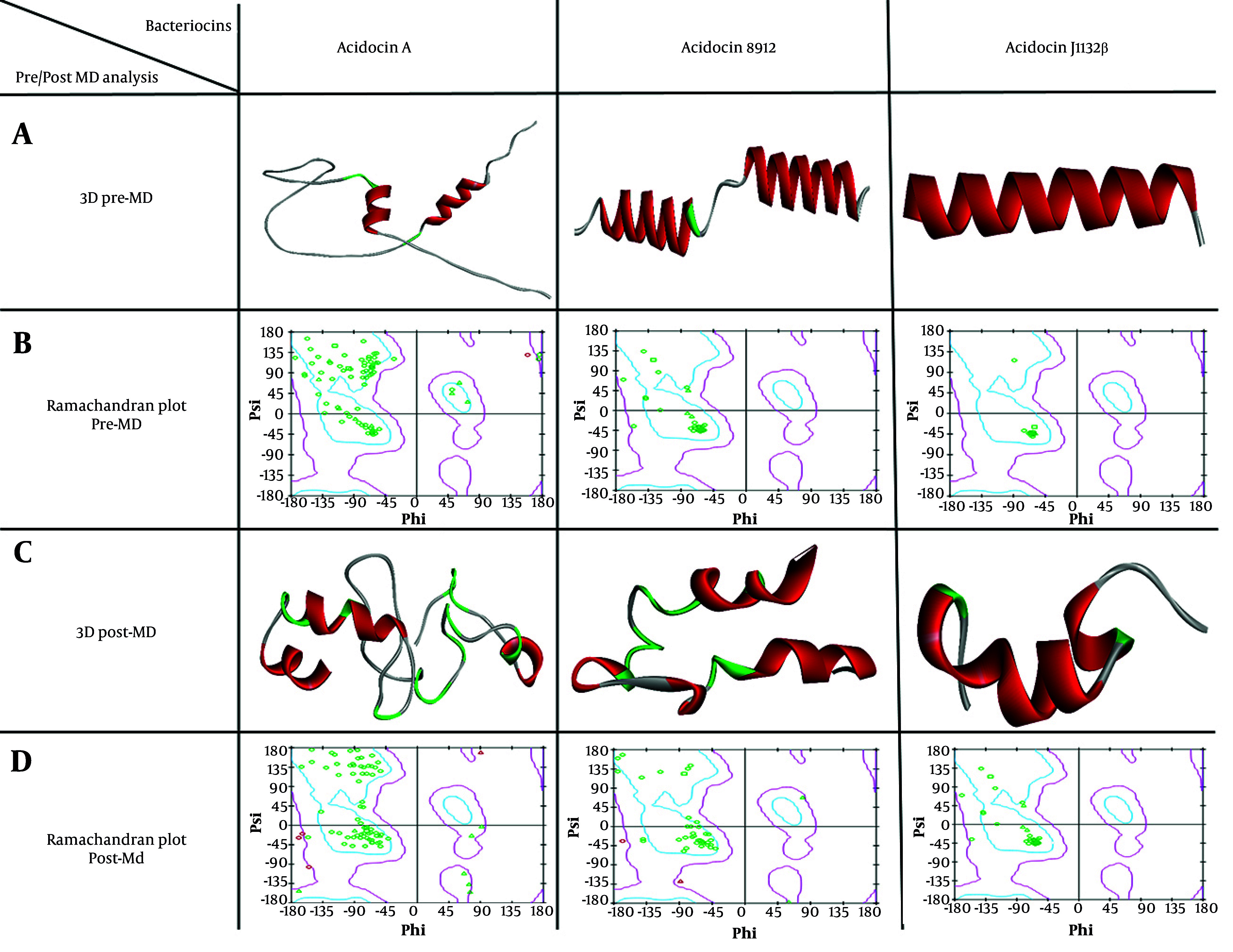
A, 3D structures of Acidocin A, acidocin 8912, and Acidocin J1132 β before molecular dynamics (MD) simulations (pre-MD), modeled using the Swiss-Model server; B, ramachandran plots corresponding to the Pre-MD structures, confirming their high quality; C, 3D structures of the peptides after MD simulations (post-MD), showing significant conformational changes; D, post-MD Ramachandran plots, illustrating the stabilization of the peptides in their most energetically favorable states.

The sequences of acidocin J1132 β, acidocin 8912, and acidocin A underwent homology modeling using the Swiss-Model server, yielding their respective PDB structures. As displayed in Figure 1, Ramachandran plot analysis conducted prior to MD simulations affirmed the high stereochemical quality of these initial models. To further refine these structural models and attain energetically favorable conformations with enhanced reliability, MD simulations were subsequently performed. Figure 1 illustrates the significant 3D conformational transitions observed in the peptides post-MD simulation, indicating their stabilization in energetically minimized states throughout the simulation process. The final optimized structures, outputted in Gromos87 (GRO) format, were then converted to PDB files to facilitate downstream molecular docking investigations.

### 4.2. Molecular Docking Reveals Specific Binding Patterns of Acidocin to Receptor Tyrosine Kinase-Like Orphan Receptor 1

Docking studies were performed to investigate the interactions between ROR-1 and the bacteriocins acidocin J1132 β, acidocin 8912, acidocin A, and acidocin B. These simulations were conducted utilizing the HDOCK server (18). The HDOCK server employs a sophisticated hybrid algorithm that integrates both template-based and template-free docking methodologies. This approach allows for the utilization of diverse input data, including amino acid sequences and experimentally determined 3D structures deposited in the PDB. Table 2 presents the computed binding affinities, expressed as scores, for the interactions of ROR-1 with acidocin A, acidocin B, acidocin 8912, and acidocin J1132 β. The respective binding scores are -231.74, -210.07, -201.70, and -170.81, respectively. These negative scores indicate thermodynamically favorable binding events, suggesting a propensity for complex formation. Notably, acidocin A exhibits the most negative binding score, signifying the strongest predicted interaction with the ROR-1 protein.

**Table 2.** Molecular Docking Results of Receptor Tyrosine Kinase-Like Orphan Receptor 1 Receptor-Bacteriocins Interactions

Bacteriocin	Binding Score (kcal/mol)
**Acidocin A**	-231.74
**AcidocinB**	-210.07
**Acidocin8912**	-201.70
**Acidocin J1132 β**	-170.81

**Figure 2.**
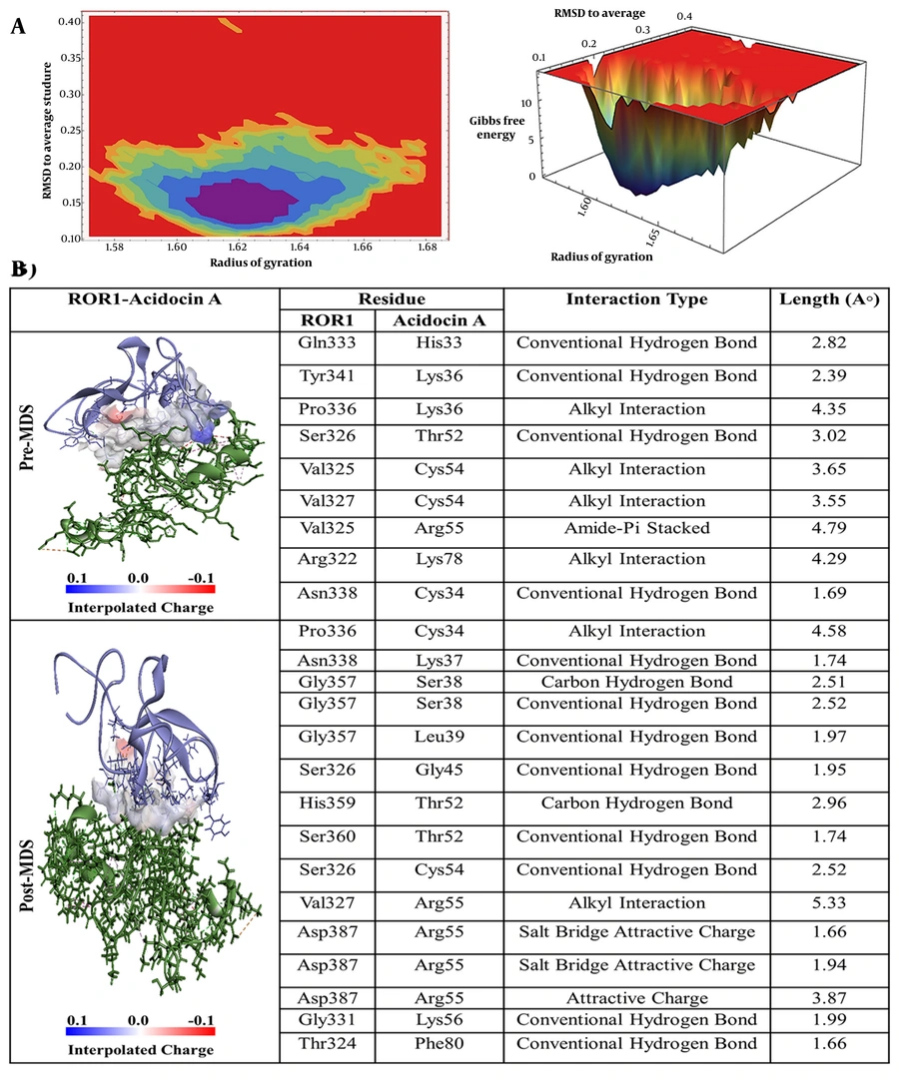
A, Free energy landscape (FEL) analysis for the receptor tyrosine kinase-like orphan receptor 1 (ROR1)-Acidocin A complex, showing the most stable conformational states during molecular dynamics (MD) simulations; B, interaction details of Acidocin A with ROR1 before (pre-MD) and after (post-MD) MD simulations.

Based on the detailed interaction analysis presented in Figure 2B, several crucial contact points were identified between acidocin A and ROR-1. Specifically, histidine at position 33 (His33) established a classical hydrogen bond with glutamine at position 333 (Gln333). Lysine at position 36 (Lys36) engaged in a hydrogen bond with tyrosine at position 341 (Tyr341) and an alkyl interaction with proline at position 336 (Pro336) of ROR-1. Threonine at position 52 (Thr52) established a hydrogen bond with serine at position 326 (Ser326), while cysteine at position 54 (Cys54) participated in alkyl interactions with valine at positions 325 (Val325) and 327 (Val327). Furthermore, arginine at position 55 (Arg55) exhibits an amide-π stacking interaction with Val325, and lysine at position 78 (Lys78) forms an alkyl interaction with arginine at position 322 (Arg322) of ROR-1.

**Figure 3.**
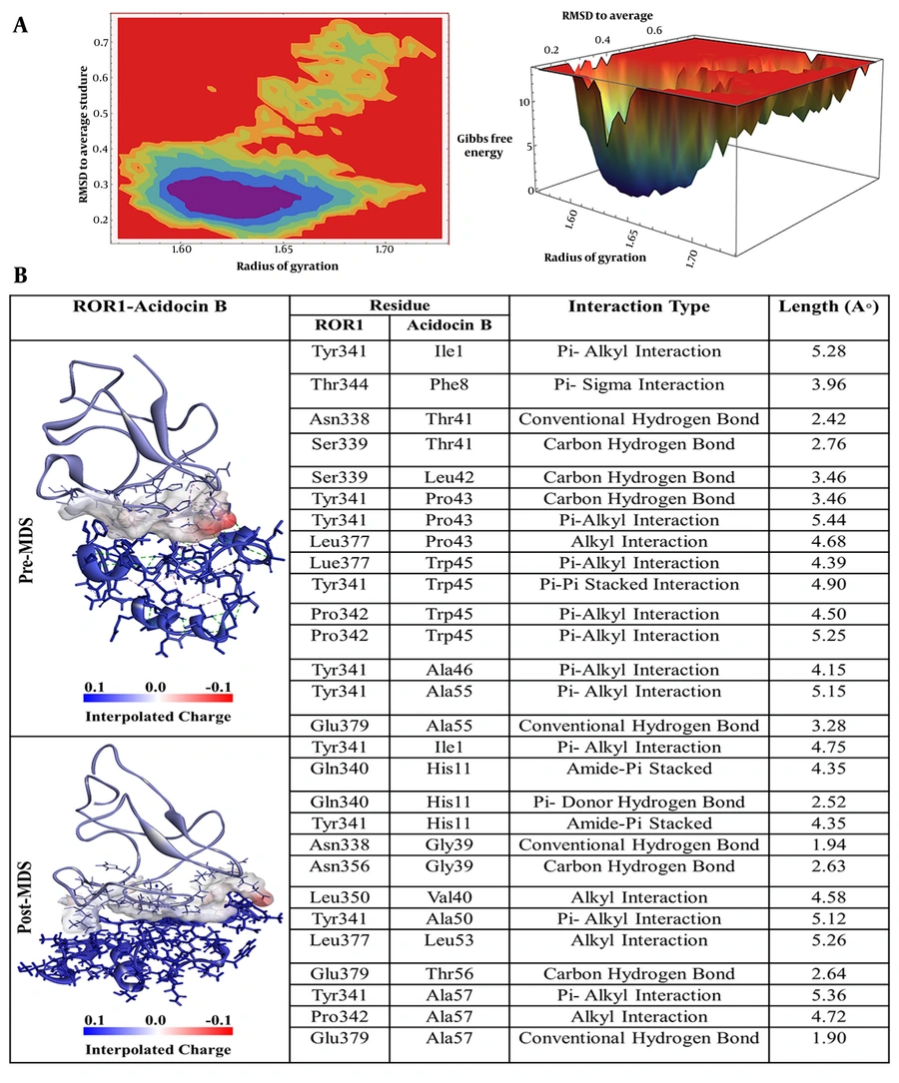
A, Free energy landscape (FEL) analysis for the receptor tyrosine kinase-like orphan receptor 1 (ROR1)-Acidocin B complex, showing the most stable conformational states during molecular dynamics (MD) simulations; B, interaction details of Acidocin B with ROR1 before (pre-MD) and after (post-MD) MD simulations.

The peptide Acidocin B, which demonstrated the second strongest binding affinity, displayed a multitude of noteworthy interactions with ROR-1, as illustrated in Figure 3B. Specifically, isoleucine at position 1 (Ile1) of acidocin B formed a π-alkyl interaction with the Tyr341 of ROR-1. Furthermore, phenylalanine at position 8 (Phe8) of the peptide engaged in a π-sigma interaction with threonine at position 344 (Thr344) of the receptor. Significantly, threonine at position 41 (Thr41) of acidocin B established two hydrogen bonds with asparagine at position 338 (Asn338) and serine at position 339 (Ser339) of ROR-1. Leucine at position 42 (Leu42) of acidocin B and proline at position 43 (Pro43) played a crucial role by forming both hydrogen and π-alkyl bonds with Tyr341 and an alkyl interaction with leucine at position 377 (Leu377) of ROR-1. Tryptophan at position 45 (Trp45) of acidocin B interacted via π-alkyl interactions with Leu377 and proline at position 342 (Pro342) of ROR-1, as well as a π-π stacked interaction with Tyr341. Finally, alanine at positions 46 and 55 (Ala46 and Ala55) of acidocin B formed π-alkyl interactions with Tyr341, and Ala55 also established a conventional hydrogen bond with glutamic acid at position 379 (Glu379) of ROR-1.

**Figure 4.**
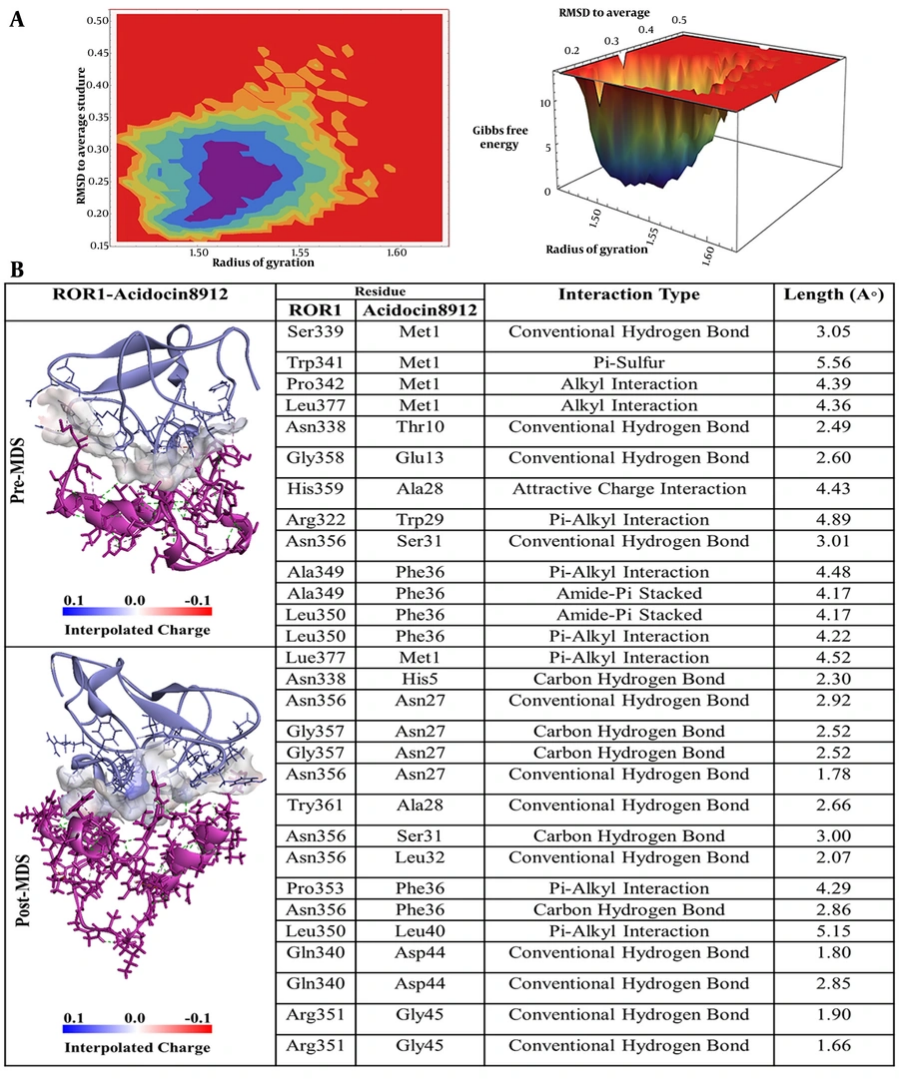
A, Free energy landscape (FEL) analysis for the receptor tyrosine kinase-like orphan receptor 1 (ROR1)-Acidocin 8912 complex, showing the most stable conformational states during molecular dynamics (MD) simulations; B, interaction details of Acidocin 8912 with ROR1 before (pre-MD) and after (post-MD) MD simulations.

Referring to acidocin 8912, as depicted in Figure 4B, methionine at position 1 (Met1) established a conventional hydrogen bond with Ser339 of ROR-1. Furthermore, Met1 exhibited a π-sulfur interaction with tryptophan at position 341 (Trp341) and two alkyl interactions with Pro342 and Leu377. In addition, threonine at position 10 (Thr10) formed a hydrogen bond with Asn338, and glutamic acid at position 13 (Glu13) engaged in a conventional hydrogen bond with glycine at position 358 (Gly358) of ROR-1. Alanine at position 28 (Ala28) displayed an attractive charge interaction with histidine at position 359 (His359), while tryptophan at position 29 (Trp29) participated in a π-alkyl interaction with Arg322. Serine at position 31 (Ser31) formed a hydrogen bond with asparagine at position 356 (Asn356), and phenylalanine at position 36 (Phe36), identified as a key residue, established both π-alkyl and amide-π stacked interactions with alanine at position 349 (Ala349) and leucine at position 350 (Leu350) of ROR-1.

**Figure 5.**
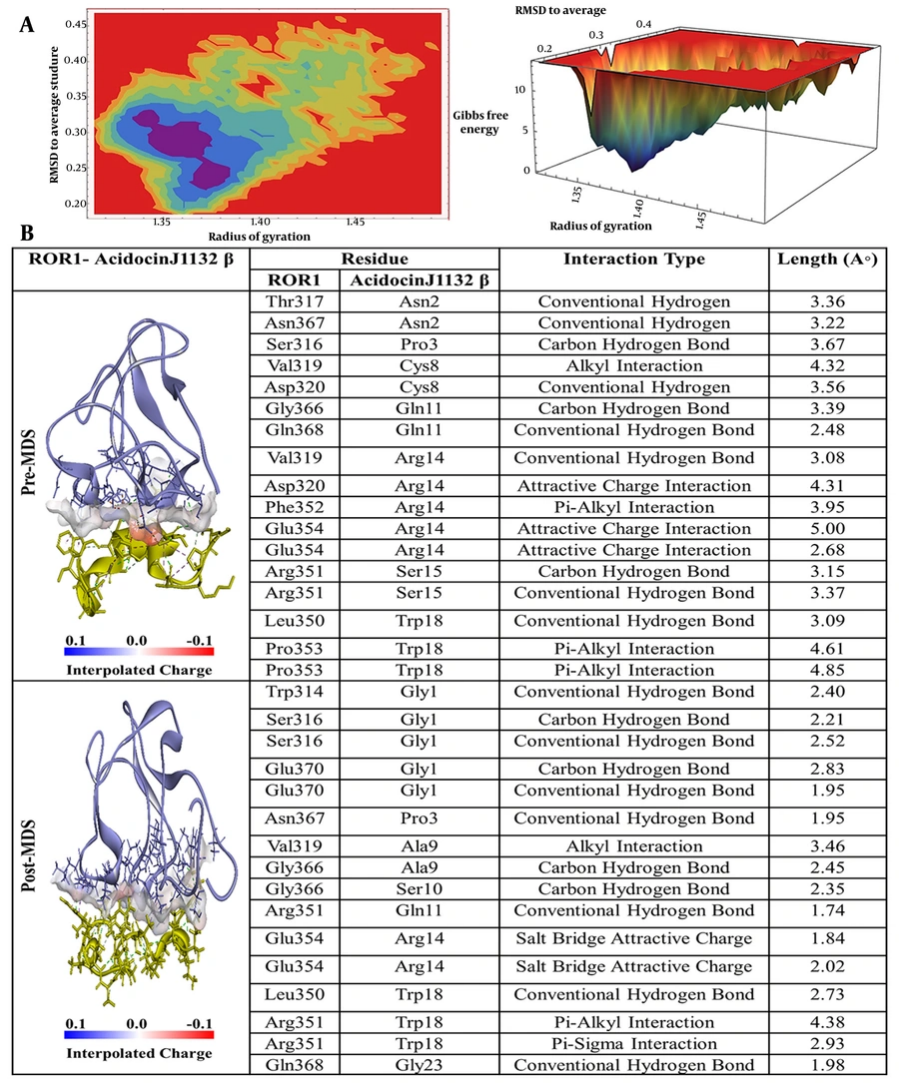
A, Free energy landscape (FEL) analysis for the receptor tyrosine kinase-like orphan receptor 1 (ROR1)-Acidocin J1132 β complex, showing the most stable conformational states during molecular dynamics (MD)simulations; B, interaction details of Acidocin J1132 β with ROR1 before (pre-MD) and after (post-MD) MD simulations.

In the case of acidocin J1132 β, as depicted in Figure 5B, asparagine at position 2 (Asn2) formed two conventional hydrogen bonds with threonine at position 317 (Thr317) and asparagine at position 367 (Asn367) of the ROR-1 protein. The proline at position 3 (Pro3) was involved in a carbon-hydrogen bond with serine at position 316 (Ser316), while cysteine at position 8 (Cys8) established an alkyl interaction with valine at position 319 (Val319) and a hydrogen bond with aspartic acid at position 320 (Asp320). Furthermore, glutamine at position 11 (Gln11) formed two hydrogen bonds with glycine at position 366 (Gly366) and glutamine at position 368 (Gln368). Notably, arginine at position 14 (Arg14) exhibited five interactions with ROR-1, including a hydrogen bond with Val319, a π-alkyl interaction with Phe352, and three attractive charge interactions with Asp320 and glutamic acid at position 354 (Glu354). Ser15 formed two hydrogen bonds with arginine at position 351 (Arg351), and tryptophan at position 18 (Trp18) established a hydrogen bond with Leu350 and two π-alkyl interactions with proline at position 353 (Pro353).

### 4.3. Results from Molecular Dynamics Simulations Indicate That Acidocin Promotes Notable Conformational Alterations

Root mean square deviation (RMSD) serves as a prevalent quantitative metric in MD simulations for evaluating the temporal stability and conformational transitions of biomolecular systems, specifically proteins and their ligand complexes (32). Generally, lower RMSD values indicate a more structurally consistent system throughout the simulation, whereas higher values suggest significant structural fluctuations or pronounced conformational changes. As illustrated in Figure S1-A in Supplementary File, the RMSD of the ROR-1 protein in its unbound state remains consistently low and stable over the course of the simulation, with the calculated values staying below 0.3 nm. The RMSD of the acidocin J1132 β-ROR-1 complex (indicated in yellow) initiates within the range of 0.4 to 0.5 nm and exhibits a progressive increase, punctuated by discernible oscillations throughout the simulation timeline. Approaching 80 ns, the complex attains a maximum RMSD value, signifying substantial alterations in its conformation. Nevertheless, the general trend observed is analogous to that of acidocin 8912, implying a moderate degree of stability characterized by intermittent phases of conformational readjustment. Conversely, the RMSD trajectory for the acidocin 8912-ROR1 complex (depicted in purple) reaches its apex at approximately 0.5 nm around the 27 ns mark, after which it demonstrates a stabilization. The overall RMSD profile exhibits a trend analogous to that observed for acidocin J1132 β, characterized by moderate fluctuations, which suggests that acidocin 8912 induces alterations in the molecular conformation that subsequently reach a stable state. Notably, the RMSD values for the acidocin A-ROR-1 complex (represented in green) remain comparatively low, ranging from 0.4 to 0.5, indicating a lower degree of structural dynamics relative to the systems depicted by the yellow and purple lines. This observation implies that acidocin A establishes a more consistent interaction with ROR-1, resulting in fewer conformational alterations within the receptor and thus forming a more stable complex. Conversely, the RMSD values for the acidocin B-ROR-1 complex (indicated in blue) are the highest among the complexes examined, reaching up to 0.9 nm. This elevated RMSD suggests that acidocin B induces significant structural rearrangements in ROR-1, indicating a more dynamic and less stable interaction compared to the other peptides under investigation. The RMSF profile for the isolated ROR-1 protein (depicted in black in Figure S1-B in Supplementary File) demonstrates consistently low values across all amino acid residues. This observation signifies a structurally stable protein with minimal internal dynamics and conformational flexibility. In contrast, the formation of the acidocin J1132 β-ROR-1 complex (illustrated in yellow) results in a notable increase in residue fluctuations, particularly within the region spanning residues 370 to 400. This augmented flexibility suggests that binding of acidocin J1132 β induces significant conformational alterations in these specific segments of the ROR-1 protein. These findings imply a dynamic interaction between the two molecules, which consequently affects the overall stability of the resulting complex. The RMSF profile of the acidocin 8912-ROR-1 complex (illustrated in purple) exhibits pronounced peaks in the vicinity of residues 390 and 430. These regions of heightened flexibility are analogous to those observed in the acidocin J1132 β. While the presence of these fluctuations suggests that acidocin 8912 binding elicits significant conformational alterations within the complex, the recurrence of similar flexibility patterns implies the existence of shared regions that undergo dynamic changes upon peptide binding. The acidocin A-ROR-1 complex (represented in green) exhibits consistently reduced RMSF values, particularly within the amino acid sequence ranges of 370 - 400 and 420 - 450, relative to the comparative complexes. This observation suggests that acidocin A establishes a more stable and structurally constrained interaction with ROR-1. This tighter binding interface consequently results in diminished conformational dynamics within the complex and an overall enhancement of its structural integrity. The reduced RMSF values observed across these crucial regions correlate with the lowest RMSD values. This consistency substantiates the strong and stable binding of acidocin A to ROR-1. Conversely, the acidocin B-ROR-1 complex (indicated in blue) exhibits the highest RMSF values among all the complexes analyzed, particularly around amino acid residues 390 and 430. This elevated RMSF suggests that acidocin B induces significant conformational changes and increased flexibility within the ROR-1 protein structure. This implies a more dynamic and, consequently, a less stable interaction in comparison to the other peptides investigated. These RMSF findings provide additional corroborative evidence supporting the conclusions drawn from the RMSD analysis and molecular docking studies. The radius of gyration (Rg), as illustrated in Figure S1-C in Supplementary File, serves as a metric within MD simulations to quantify the spatial extent or compactness of a protein or protein-ligand complex. Specifically, Rg indicates how the molecule’s mass is distributed relative to its center of mass. A lower Rg value generally signifies a more densely packed or globular structure, whereas a higher Rg value suggests a more extended or less compact conformational state. The Rg for unbound ROR-1 (indicated in black) exhibits consistent and low values, approximately 1.3 nm, throughout the simulation period. This observation suggests that in the absence of ligand binding, ROR-1 adopts and maintains a compact and stable 3D architecture, indicative of its intrinsic structural stability. Conversely, the acidocin J1132–β-ROR-1 complex (represented in yellow) demonstrates slightly elevated Rg values, which stabilize around 1.45 nm. This suggests that the complex exhibits relative stability but displays a slightly more expanded conformation compared to ROR-1 in its unbound state. This observation implies a moderate level of stability accompanied by some structural rearrangements upon acidocin J1132 β binding. The Rg for the acidocin 8912-ROR-1 complex (indicated in purple) shows values around 1.5 nm, which is similar to that of acidocin J1132 β alone. The fluctuations observed are moderate, suggesting a stable complex; however, the increased conformational changes compared to ROR-1 alone suggest an intermediate level of overall stability. The acidocin A-ROR-1 complex (depicted in green) exhibits Rg values in the approximate range of 1.55 to 1.6 nm. Notably, despite these elevated values, this complex demonstrates fewer fluctuations in its Rg compared to the majority of other complexes examined, with the exception of the unbound ROR-1 protein. This observation suggests that acidocin A promotes a more stable and compact structural arrangement upon interaction with ROR-1. This inference is further supported by RMSD and RMSF analyses, which indicated a robust and stable binding interaction. Conversely, the acidocin B-ROR-1 complex (illustrated in blue) displays the highest Rg values among all the complexes investigated, registering around 1.6 to 1.7 nm. Hydrogen bonds play a pivotal role in determining the stability and specificity of interactions between proteins and their ligands (32). As depicted in Figure S1-D in Supplementary File, MD simulations of the acidocin J1132-β-ROR-1 complex (rendered in yellow) revealed the formation of up to 12 hydrogen bonds throughout the simulation trajectory. This substantial number suggests a strong interaction, although it exhibits dynamic behavior, with periods of both hydrogen bond formation and disruption, ultimately reflecting an interaction of moderate overall stability. In contrast, the acidocin 8912-ROR-1 complex consistently formed up to 10 hydrogen bonds. This sustained hydrogen bond network suggests a stable interaction, maintaining an intermediate level of stability throughout the simulated timeframe. Notably, the acidocin A-ROR-1 complex exhibited the highest number of hydrogen bonds, reaching a maximum of 14 during the simulation. This extensive hydrogen bonding network signifies a very strong and stable interaction, consistent with prior RMSD, RMSF, and Rg analyses, which collectively identified acidocin A as the most stable and effective binding partner. Meanwhile, the acidocin B–ROR-1 complex formed a maximum of 7 hydrogen bonds. The free energy landscape (FEL) analysis pinpointed the most thermodynamically stable conformations of ROR-1 complexes when bound to acidocin J1132 β, acidocin 8912, acidocin A, and acidocin B during MD simulations (33). These conformations were subsequently subjected to post-MD analysis (Figure 2A, Figure 3A, Figure 4A, and Figure 5A). Interaction analysis conducted post-MD revealed that acidocin J1132 β established up to 11 hydrogen bonds, indicating robust interactions characterized by consistent hydrogen bonding patterns (Figure 5B). Acidocin 8912, as illustrated in Figure 4B, formed 13 hydrogen bonds, a notable increase from the number observed prior to MD, suggesting enhanced stability following the simulation. While acidocin A exhibited a maximum of 11 hydrogen bonds post-MD (Figure 2B), representing a significant rise from the 3 hydrogen bonds observed pre-MD, highlighting its strong binding affinity. In contrast, acidocin B formed only 4 hydrogen bonds after MD, a reduction compared to its pre-MD count, suggesting a lower degree of stable interactions (Figure 3B). To further substantiate these findings, we applied the formula G₂ - G₁ = Kb ln(qi/qmax), which quantitatively relates the depth of the energy well to conformational flexibility and inertia. According to this analysis, deeper wells correspond to more stable conformations, confirming that acidocin A forms the most stable complex with ROR-1, whereas acidocin B remains comparatively less stable. Principal component analysis (PCA), a statistical methodology designed for the reduction of dimensionality in intricate datasets while preserving the principal sources of variance, was implemented in this investigation (34). Specifically, PCA was applied to the MD simulation trajectories of ROR-1 complexes in association with acidocin J1132 β, acidocin 8912, acidocin A, and acidocin B. As depicted in Figure S2-B in Supplementary File, the conformational landscapes explored by these complexes were analyzed through projections onto the initial two principal components (PCs: PC1 and PC2). The acidocin J1132 β-ROR-1 complex exhibits a considerable dispersion across the conformational space, with discernible clusters representing energetically favorable states and more sparsely distributed points signifying transitional conformations. This observation suggests an equilibrium between structural stability and dynamic flexibility, indicative of moderate overall stability coupled with conformational adaptability. Analogously, the acidocin 8912-ROR-1 complex demonstrates substantial fluctuations along both PC1 and PC2. The presence of clusters suggests relatively stable conformational basins, while the interspersed points denote transitions between these states. This implies that acidocin 8912 maintains a stable interaction with ROR-1 while concurrently permitting requisite conformational plasticity. The acidocin A-ROR-1 complex exhibits tightly clustered data points, indicating a highly stable interaction characterized by minimal conformational flexibility. This compact distribution suggests that acidocin A maintains a rigid binding mode with ROR-1, which supports its strong binding affinity and overall stability. Conversely, the acidocin B-ROR-1 complex displays a broader dispersion of data points, signifying greater flexibility and comparatively lower stability than the acidocin A complex. These scattered points reflect a more dynamic binding mechanism involving substantial conformational changes, implying a less stable interaction. As depicted in Figure S2-A in Supplementary File, Porcupine plots are employed to visualize the direction and magnitude of the principal component motions identified through PCA. Within these plots, each arrow represents the directional movement of a specific residue within the protein, with the length of the arrow proportional to the extent of that movement. This visual representation facilitates the understanding of protein regions undergoing significant conformational alterations during the simulation. The acidocin J1132 β-ROR1 complex displays considerable mobility across various structural domains, as evidenced by multiple long vectors oriented in diverse directions, indicative of substantial conformational transitions. Notably, four extended projections are observed at the terminal regions, exhibiting a directional bias opposite to the main protein body. Furthermore, the protein core features moderate projections that also demonstrate opposing directional dynamics relative to the termini. Together, these observations suggest that acidocin J1132 β induces significant dynamic conformational changes, oscillating between states of stability and flexibility, thereby conferring notable conformational adaptability to the complex. The acidocin 8912-ROR-1 complex exhibits notable dynamic behavior, as evidenced by four extended vectors at its distal ends, signifying substantial mobility in these regions. The spatial arrangement of these vectors contrasts with that observed in J1132. Conversely, the protein's central domain displays minimal vector representation, implying a structurally stable core juxtaposed with highly flexible terminal segments. This configuration, characterized by a rigid central region and mobile termini, suggests that acidocin 8912 can sustain stable interactions with ROR-1 while simultaneously accommodating essential conformational plasticity within its peripheral domains. The acidocin A-ROR-1 complex exhibits limited conformational flexibility, as indicated by several moderate-intensity peaks at the protein's termini and a scarcity of vectors within its main structure. This suggests that the interaction between acidocin A and ROR-1 results in a highly stable complex, leading to rigidification of the protein conformation. The observed lack of significant internal motion within the protein underscores the strong and stable binding affinity of acidocin A for ROR-1, thereby supporting its potential as a promising therapeutic agent.

### 4.4. Binding Free Energy Calculations Based on the Molecular Mechanics/Poisson-Boltzmann Surface Area Approach Reveal That Acidocin a Demonstrates the Highest Affinity for the Receptor Tyrosine Kinase-Like Orphan Receptor 1

To gain a comprehensive understanding of the interaction energies between ROR-1 and the peptides, Molecular Mechanics-Poisson Boltzmann Surface Area (MM-PBSA) calculations were performed on the final 1 ns of the MD trajectory. This analysis aimed to estimate the binding free energy ΔG of the protein-ligand complexes and to pinpoint key amino acid residues that significantly contribute to these binding interactions. The calculated ΔTOTAL for each complex were as follows: Acidocin A-ROR-1: -42.34 kcal/mol, acidocin B-ROR-1: -38.96 kcal/mol, acidocin 8912-ROR-1: -41.68 kcal/mol, and acidocin J1132-ROR-1: -37.77 kcal/mol. These findings suggest that acidocin A exhibits the highest binding affinity for ROR-1, closely followed by acidocin 8912, while acidocin B and acidocin J1132 display slightly weaker binding affinities.

As depicted in Figure S3-A in Supplementary File, the acidocin A-ROR-1 complex is characterized by key residues that significantly contribute to the energetic stability of the interaction. These residues include threonine at position 324 (Thr324), Gln333, tyrosine at position 361 (Tyr361), and His359 within the ROR-1 protein, as well as leucine at position 39 (Leu39) and Thr52 of acidocin A. In the case of the acidocin B-ROR-1 complex, Figure S3-B in Supplementary File illustrates that Tyr341 of ROR-1 and histidine at position 11 (His11) of acidocin B are the primary residues involved in significant energetic contributions. For the acidocin 8912-ROR-1 complex, as shown in Figure S3-C in Supplementary File, the crucial residues for energetic interactions are Leu350 and Arg351 of ROR-1 and Phe36 of acidocin 8912. Finally, analysis of the acidocin J1132 β-ROR1 complex, presented in Figure S3-D in Supplementary File, reveals that Glu354 and Arg351 of ROR-1, along with Arg14 and Trp18 of acidocin J1132 β, are the key residues mediating significant energetic contributions to the complex formation.

### 4.5. Outcomes from the Steered Molecular Dynamics Simulations

The steered molecular dynamics (SMD) simulation analysis conducted to quantify the pulling forces of the four ROR-1 complexes yields significant insights into their binding stabilities. Specifically, the force-time profiles generated from these simulations offer a detailed representation of the resistance encountered by each complex throughout the pulling process, thereby serving as an indicator of their respective binding strengths and overall stability.

Each SMD simulation was performed in GROMACS 2022.06 using the pull code module. The peptide molecule was steered along the z-axis (pull-coord1-dim = N N Y) while restraining the protein backbone. A harmonic spring constant of 1000 kJ/mol/nm² was applied with a pulling velocity of 0.1 nm/ps. Preliminary trials demonstrated that complete peptide dissociation typically occurred within 300 - 400 ps; therefore, the simulation time was set to 500 ps to ensure full separation. The force-time profiles obtained over this 0 - 500 ps interval were subsequently analyzed to quantify the maximum unbinding forces of each complex.

As depicted in Figure S4 in Supplementary File, the acidocin A-ROR-1 complex exhibits a peak pulling force of approximately 700 kJ/mol/nm. This maximum force is observed initially followed by a gradual decline beginning around 300 ps. This observation highlights the robust mechanical stability of the Acidocin A-ROR-1 interaction under external force. The acidocin B-ROR-1 complex exhibited a peak force of approximately 350 kJ/mol/nm in SMD simulations, indicating a lower resistance to dissociation during the pulling process, indicating a weaker mechanical resistance to unbinding compared to the other complexes. The acidocin 8912-ROR-1 complex displayed an intermediate peak unbinding force of approximately 500 kJ/mol/nm. This moderate level of mechanical resistance indicates a binding stability that is lower than Acidocin A but still represents a significant interaction within the complex. Finally, the acidocin J1132 β-ROR-1 complex exhibited the highest peak unbinding force, reaching approximately 580 kJ/mol/nm. This substantial resistance to mechanical disruption indicates that the J1132 β complex possesses strong mechanical stability, though not as pronounced as that observed for Acidocin A.

### 4.6. The Results of Biological Studies: The Mixture of Acidocins Exhibits Cytotoxic Effects on Breast Cancer Cell Lines

Our primary goal was to assess the biological activity of the CA mixture derived from *L. acidophilus*. To evaluate its effectiveness, we determined the half-maximal inhibitory concentration (IC_50_) using the MTT assay, which is a standard method for measuring cell viability and cytotoxicity. This allowed us to identify the appropriate effective dosage of the CA mixture for further investigations.

The IC_50_ of crude acidocin (containing acidocin A) was evaluated on human BC cell lines (MCF7 and MDA-MB-231) using the MTT assay. The resulting data were analyzed, with IC_50_ values expressed in µg/mL. This concentration represents the half-maximal inhibitory concentration required to reduce cell viability by 50%. As depicted in Figure S5 in Supplementary File, the CA exhibited a significant cytotoxic effect on both MCF7 and MDA-MB-231 cell lines, as indicated by the determined IC_50_ concentration. Following a 48-hour incubation period at 37°C, the IC_50_ of the CA was established to be 867 µg/mL for MCF7 and 1 mg/mL for MDA-MB cell lines.

### 4.7. Crude Acidocin Treatment Reduces Receptor Tyrosine Kinase-Like Orphan Receptor 1-Src Signaling Activity in Breast Cancer Cells

We hypothesized that acidocin, a bacteriocin produced by *L. acidophilus*, indicates an inhibitory interaction with ROR-1 in BC cell lines. Our theory is that the levels of phosphorylated c-Src protein would decrease if the ROR-1 receptor is inhibited by the mixture of acidocins containing acidocin A. The c-Src protein serves as a substrate for ROR1. Upon activation of ROR1, there is an increase in the phosphorylated form of c-Src protein. Conversely, when ROR1 is inhibited, the levels of phosphorylated c-Src protein are diminished.

To investigate the impact of crude acidocin on ROR-1 signaling pathways, we assessed the protein levels of Src and phosphorylated Src (Src-p) using Western blot analysis. Src, a non-ROR1 implicated in tumor growth, represents a crucial factor in cancer biology. It functions as a substrate for ROR-1, and its activation through phosphorylation events mediated by ROR-1 can significantly influence the behavior of cancer cells. The ROR-1-Src signaling pathway is a critical focus in tumor biology research, with implications for developing new cancer therapies. To investigate the impact of acidocins-containing CA on this pathway, we assessed the levels of phosphorylated Src in MDA-MB-231 and MCF-7 cell lines following treatment with CA at its IC_50_ concentration. As depicted in Figure S6 in Supplementary File, Western blot analysis revealed a significant reduction in the phosphorylated Src protein in the CA-treated cell populations compared to the control group. This observation aligns with our computational investigations, which indicated an inhibitory action of acidocin on ROR-1. The results propose that a compound (CA) extracted from *L. acidophilus* possesses the capacity to impede the ROR-1-Src signaling pathway. However, a limitation of the present study resides in the unrefined state of the bacteriocin containing acidocin A and acidocin B. To advance this line of inquiry, subsequent research should focus on the isolation of individual acidocin variants and their subsequent application to tumor cell lines.

## 5. Discussion

The antimicrobial activity of bacteriocins, such as acidocins, involves mechanisms like membrane disruption and binding to bacterial receptors. These mechanisms theoretically extend to interactions with mammalian proteins, such as ROR-1 (35). For example, enterocins produced by lactic acid bacteria have exhibited substantial interactions with bacterial membranes, leading to cytoplasmic leakage and subsequent cell death (36). This observation suggests a potentially analogous mechanism in the context of ROR-1 interactions (9, 37, 38). Moreover, molecular docking investigations involving proteins targeting cancer receptors have revealed comparable binding modes and affinities, underscoring the therapeutic significance of such interactions (39, 40).

The aforementioned interactions underscore the heterogeneity of binding mechanisms, encompassing hydrogen bonding, hydrophobic interactions, and π-interactions, which are integral to the stability and specificity of peptide-protein complexes. Congruent evidence from recent investigations emphasizes the pivotal role of these interactions in augmenting the binding affinity and functional stability of peptide-based therapeutics (41, 42).

The interaction profile of acidocin A with ROR-1, characterized by a multiplicity of hydrogen bonds and hydrophobic contacts, indicates its robust binding affinity and stability, thereby further substantiating its potential as a salient candidate for therapeutic intervention targeting ROR-1. Furthermore, the consistency observed in binding mechanisms across diverse protein targets lends support to the hypothesis that acidocin may serve as an efficacious therapeutic agent, particularly in targeting proteins such as ROR-1 implicated in pathological states (15).

The RMSD data suggest that ROR-1 exhibits an inherently structurally stable conformation in the absence of ligand binding, as indicated by previous research (43). Consequently, while all tested peptides demonstrate effective interaction with ROR-1, their impact on stability and conformation varies, which has implications for their therapeutic potential. Notably, acidocin A stands out as a promising candidate for ROR-1-targeted therapy due to its enhanced stability upon binding. The RMSD findings derived from MD simulations corroborate the results obtained from molecular docking, demonstrating that acidocin A displays the most stable interaction with ROR-1. This is evidenced by its lower RMSD values and the most negative binding score, signifying a strong binding affinity. These observations are consistent with other studies, thereby validating the reliability of MD simulations in confirming docking outcomes (44, 45).

The RMSF analysis substantiates the intrinsic structural robustness of ROR-1 when unbound to a peptide ligand. Specifically, the termini exhibit elevated flexibility, a frequently observed phenomenon in investigations of protein dynamics (33, 46, 47).

In the context of MD simulations, the temporal analysis of hydrogen bond counts yields crucial information regarding the robustness and longevity of these interactions, which are fundamental in assessing the stability of protein-ligand complexes (48, 49). The hydrogen bond analysis corroborates the findings obtained from RMSD, RMSF, and Rg analyses, as well as docking studies, underscoring that acidocin A establishes the most stable and persistent hydrogen bond network with ROR-1. This stability is manifested by the greater number of hydrogen bonds sustained throughout the simulation period. Conversely, acidocin B forms the fewest hydrogen bonds, suggesting a more labile and less stable interaction. These observations further substantiate the potential therapeutic efficacy of acidocin A in targeting ROR-1, given that its stable binding profile is critical for the effective inhibition or modulation of the receptor's function.

The findings from the FEL analysis are consistent with the RMSD, RMSF, and Rg analyses. These collective results highlight the superior conformational stability of acidocin A and the more dynamic, thus less stable, binding mode of acidocin B. Consequently, these outcomes underscore the potential of acidocin A as a promising therapeutic agent targeting ROR-1, attributed to its robust and stable interaction profile. The integrated application of molecular docking and MD simulations provides a thorough understanding of intermolecular interaction dynamics, which is crucial for the rational design of effective inhibitors against cancer-related receptors (50). This methodological approach emphasizes the significance of incorporating both static and dynamic analyses in the comprehensive evaluation of potential therapeutic candidates.

Within the framework of MD simulations, PCA is employed to dissect the essential dynamics and conformational transitions of protein-ligand complexes. This methodology entails the computation of PCs, which are eigenvectors that delineate the axes of maximal data variance. The initial two PCs (PC1 and PC2) characteristically encapsulate the most substantial conformational alterations, thereby offering valuable perspectives into the dominant motions exhibited by the protein complex (34).

The PCA outcomes reveal a wider dispersion of vectors for the acidocin B-ROR-1 complex, exhibiting characteristics akin to acidocin 8912 yet distinguished by extended vector lengths. This broad distribution is indicative of enhanced flexibility and a more dynamic structural profile in comparison to the other investigated complexes. The presence of longer vectors at the termini, coupled with a scattered distribution of vectors across the protein structure, implies that binding of acidocin B induces substantial conformational alterations within ROR-1, consequently leading to a less stable interaction. The augmented flexibility observed in this complex underscores its diminished stability relative to the acidocin A complex.

A comparative analysis of these pivotal amino acid residues with the interaction profiles derived from pre- and post-MD simulations, as well as MM-PBSA calculations, demonstrates notable congruences. For instance, within the acidocin A-ROR-1 complex, the residues Gln333 and Thr52 of ROR-1 exhibit significant interactions prior to MD simulation, a characteristic that is sustained in both the post-MD analysis and the energetic contributions identified through MM-PBSA. Similarly, in the acidocin 8912-ROR-1 complex, the pre-MD interactions involving Asn338 and Asn356 correlate with their energetic importance as determined by the MM-PBSA method. Furthermore, the Arg351 residue of ROR-1 consistently emerges as significant in both the pre- and post-MD analyses for the acidocin 8912 and acidocin J1132 β complexes, underscoring its critical role in the stability of the binding interface.

These findings underscore the dynamic nature inherent in protein-ligand interactions, emphasizing the necessity of integrating docking studies with MD simulations and MM-PBSA analysis to achieve a thorough mechanistic understanding of binding events (51). The synergistic application of MM-PBSA assessment with MD simulations provides a robust methodological framework for evaluating the energetic landscape of protein-ligand complexes and pinpointing key interacting residues. Notably, acidocin A exhibited the highest binding affinity for ROR-1, a result substantiated by significant energetic contributions from crucial amino acid residues identified consistently across both pre- and post-MD analyses. These detailed insights hold substantial implications for the rational design of effective inhibitors targeting receptors implicated in cancer, thereby highlighting the critical importance of in-depth energetic and dynamic analyses in the development of therapeutic agents.

The SMD simulation results demonstrate a strong correlation with the binding energy calculations derived from MM-PBSA analysis. Specifically, acidocin A exhibited the highest resistance to the applied pulling force, indicative of its superior binding stability, followed in descending order by acidocin J1132 β, acidocin 8912, and acidocin B. This integrated analysis, encompassing both the energetic and mechanical dimensions of the binding interactions, provides a robust understanding of the relative stabilities among these peptide-ROR-1 complexes. Furthermore, the congruence of these findings with data obtained from pre- and post-MD simulation analyses underscores the crucial amino acid residues mediating the binding and reinforces the potential of acidocin A as a highly efficacious therapeutic agent targeting ROR-1.

Furthermore, a biological investigation was undertaken to ascertain the inhibitory effects of crude acidocin on cell proliferation and the activity of the ROR-1-Src signaling axis. The evaluation of cell proliferation demonstrated that acidocin exerts a concentration-dependent and significant cytotoxic effect on the growth of the BC cell line under study. Consistent with these observations, Avand et al. reported that bacteriocins produced by *Lactococcus lactis* exhibit a cytotoxic effect on MCF7 BC cell lines and a comparatively mild cytotoxic effect on normal human umbilical vein endothelial cells (HUVECs) (52). Although the precise molecular mechanisms underlying the anti-cancer effects of bacteriocins are yet to be fully elucidated, several potential mechanisms have been proposed in the literature. These include disruption of the cell membrane and the formation of pores, which can lead to alterations in intracellular ion concentrations, changes in transmembrane potential, and ultimately, cell death (53).

To validate our computational findings, we investigated the effect of whole extracted acidocins (containing acidocin A) on the ROR-1 receptor by assessing its influence on the phosphorylation status of its downstream substrate, the c-Src protein. Given the intrinsic tyrosine kinase activity of the ROR-1 receptor, it exerts its signaling effects through the phosphorylation-mediated activation of its substrates and downstream signaling molecules. Consequently, levels of phosphorylated c-Src protein directly correlate with ROR-1 receptor activation, whereas receptor inhibition or inactivation would result in the prevalence of the dephosphorylated form of Src. Our Western blot analysis revealed that treatment with whole acidocins culminated in a discernible decrease in the levels of phosphorylated c-Src protein in both examined cell lines. This observed reduction in phosphorylated c-Src protein suggests that acidocin A in this mixture may modulate the activation state of ROR-1, implicated in a multitude of cellular processes, encompassing apoptosis and cell proliferation.

### 5.1. Limitations

This study has several limitations that should be acknowledged:

Inability to isolate individual bacteriocins: We were unable to isolate and characterize acidocin A from the crude mixture, limiting our understanding of their specific contributions to the observed biological effects.

Funding and equipment constraints: Limited resources restricted our capacity for extensive purification and analysis, which could have provided deeper insights into the bacteriocins' mechanisms of action.

Small size of Acidocins: The very small size of acidocins posed additional challenges in purification and quantification, impacting our ability to study these compounds in detail.

In vitro nature of the study: The research was conducted in vitro, which may not fully reflect the complexity of biological systems in vivo, potentially altering the observed effects.

By recognizing these limitations, we aim to provide context for our findings and suggest avenues for future research.

### 5.2. Conclusions

This investigation underscores the therapeutic potential of bacteriocins, particularly those originating from *L. acidophilus*, in targeting ROR-1, a receptor implicated in aggressive BC. Using an integrated computational approach encompassing molecular docking, MD simulations, and MM-PBSA analyses, acidocin A was identified as the most promising candidate. This bacteriocin demonstrated the highest binding affinity and exhibited significant stability in its complex with ROR-1. The findings suggest that the interaction between acidocin A and ROR-1 may disrupt crucial signaling pathways involved in cancer cell survival, positioning it as a viable candidate for further preclinical and clinical development as an anticancer agent. Furthermore, this study emphasizes the significance of integrating computational methodologies for the exploration and validation of novel therapeutic strategies against challenging cancer targets. Our in vitro study revealed that treatment with whole acidocins exerted from *L. acidophilus*, resulted in a noticeable decrease in phosphorylated c-Src protein levels in both examined cell lines. This reduction suggests that this mixture of acidocins (containing acidocin A) may modulate the activation state of ROR-1, further supporting its role as a potential therapeutic agent. Future research endeavors should prioritize experimental validation of these molecular interactions and in vivo evaluation of acidocin A's efficacy, thereby potentially paving the way for the development of novel microbiota-derived therapies in cancer treatment.

ijpr-24-1-163509.pdf

## Data Availability

The dataset presented in the study is available on request from the corresponding author during submission or after publication.
